# Modifications to toxic CUG RNAs induce structural stability, rescue mis-splicing in a myotonic dystrophy cell model and reduce toxicity in a myotonic dystrophy zebrafish model

**DOI:** 10.1093/nar/gku941

**Published:** 2014-10-10

**Authors:** Elaine deLorimier, Leslie A. Coonrod, Jeremy Copperman, Alex Taber, Emily E. Reister, Kush Sharma, Peter K. Todd, Marina G. Guenza, J. Andrew Berglund

**Affiliations:** 1 Department of Chemistry and Biochemistry, Institute of Molecular Biology, University of Oregon, Eugene, Oregon, USA; 2 Department of Neurology, University of Michigan, Ann Arbor, Michigan, USA

## Abstract

CUG repeat expansions in the 3′ UTR of dystrophia myotonica protein kinase *(DMPK)* cause myotonic dystrophy type 1 (DM1). As RNA, these repeats elicit toxicity by sequestering splicing proteins, such as MBNL1, into protein–RNA aggregates. Structural studies demonstrate that CUG repeats can form A-form helices, suggesting that repeat secondary structure could be important in pathogenicity. To evaluate this hypothesis, we utilized structure-stabilizing RNA modifications pseudouridine (Ψ) and 2′-O-methylation to determine if stabilization of CUG helical conformations affected toxicity. CUG repeats modified with Ψ or 2′-O-methyl groups exhibited enhanced structural stability and reduced affinity for MBNL1. Molecular dynamics and X-ray crystallography suggest a potential water-bridging mechanism for Ψ-mediated CUG repeat stabilization. Ψ modification of CUG repeats rescued mis-splicing in a DM1 cell model and prevented CUG repeat toxicity in zebrafish embryos. This study indicates that the structure of toxic RNAs has a significant role in controlling the onset of neuromuscular diseases.

## INTRODUCTION

Myotonic dystrophy is a genetic disorder that is the most common adult-onset form of muscular dystrophy, affecting ∼1 in 8000 people. Myotonic dystrophy type 1 (DM1) is caused by a CTG repeat expansion mutation in the 3′ non-coding region of the dystrophia myotonica protein kinase (*DMPK*) gene. Unaffected individuals have up to 40 CTG repeats at this location, while DM1 patients have >40 and up to thousands of CTG repeat (1,2). Our current understanding of the DM1 disease mechanism is that the CTG repeats are transcribed into toxic CUG-repeat RNA, which sequesters RNA-binding proteins that are important for gene regulation (3,4).

Muscleblind-like proteins (MBNL1, MBNL2 and MBNL3) are the primary RNA-binding proteins that are sequestered to the expanded CUG repeats (5). MBNL proteins regulate alternative splicing and other RNA processing events (6). During pre-mRNA splicing, the spliceosome must choose which exons to include based on developmental or environmental signals. This is a common regulatory event that vastly diversifies the proteome by allowing multiple protein isoforms to be expressed from a single gene. MBNL proteins function by interacting with their RNA-binding sites within pre-mRNA downstream or upstream of alternatively spliced exons and direct the spliceosome to either include or exclude certain exons (7,8). Many symptoms of DM1 are caused by expression of fetal isoforms due to mis-regulated alternative splicing (9). For example, when MBNL genes are expressed in adults, they regulate the inclusion of the insulin receptor's (*INSR*) exon 11 (10). One symptom of DM1 is insulin resistance that is likely partly due to the mis-regulation of this transcript (9). Another MBNL target that is mis-regulated in DM1 is cardiac troponin T (*TNNT2*). In this case MBNL promotes the exclusion of exon 5 (11). Accordingly, one of the leading causes of death in DM1 is cardiac dysfunction, in part possibly due to mis-splicing of *TNNT2* (12,13).

MBNL proteins contain four Zn fingers that fold into two similar domains (each domain contains two Zn fingers) that bind RNA on opposing faces of the domain (14,15). The consensus MBNL binding site is YGCY (Y represents pyrimidines), which is found in its pre-mRNA targets, and several copies of this motif are usually necessary for splicing regulation by MBNL proteins (7,16). The CUG repeats are toxic because they contain hundreds or thousands of YGCY motifs repeated in the expanded CUG repeats (17). Presumably, the many MBNL proteins binding the expanded CUG repeats leads to formation of aggregated MBNL-CUG repeat foci in patient cells and model systems (18,19).

Structure-probing, crystal and nuclear magnetic resonance structures have shown that CUG repeats can form double-stranded A-form helices (20–23). A crystal structure by Teplova *et al.* showed how a single MBNL1 zinc finger domain interacts with an RNA containing a YGCY binding site primarily via the Watson–Crick face of the nucleotides (24). This result suggested that MBNL proteins will not bind or bind weakly to CUG repeats when they are in double-stranded or helical conformation, as the Watson–Crick faces of the bases form hydrogen bonds with the opposite strand, leaving them unavailable for interaction with MBNL. The resulting prediction is that if CUG repeats are stabilized in a double-stranded or helical structure, these repeats will have reduced or no toxicity because MBNL proteins will not be sequestered.

**Figure 1.**
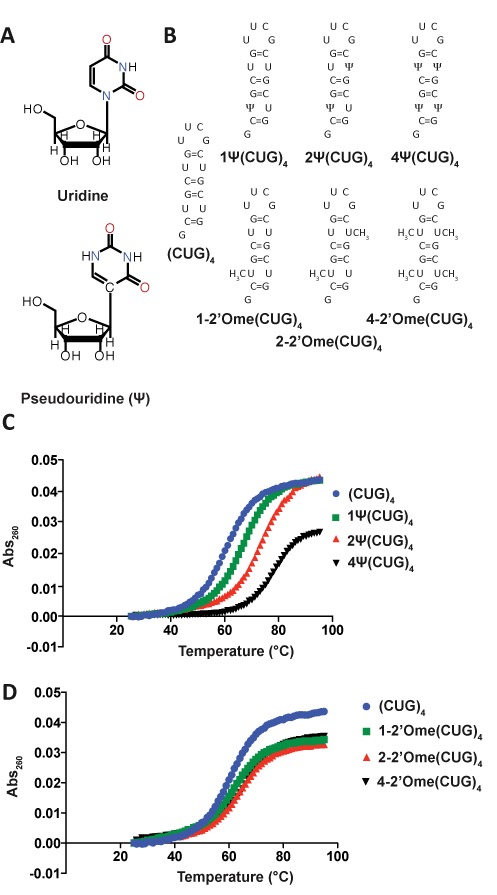
Ψ and 2′-O-methyl modifications increase the structural stability of (CUG)_4_ RNA. (**A**) Uridine and Pseudouridine. Pseudouridine (Ψ) is an isomerization product of uridine that has a C–C bond connecting the base to the ribose as well as an extra N–H bond donor. (**B**) Depictions of the structures of unmodified (CUG)_4_ RNA along with (CUG)_4_ with one, two and four Ψ and 2′-O-methyl modifications. (**C**) Melting curves for (CUG)_4_ RNAs modified with Ψ. The total change in melting temperature between unmodified (CUG)_4_ and 4Ψ(CUG)_4_ is ∼20°C (59±1, 67±2, 72±1 and 78±1°C for (CUG)_4_, 1Ψ(CUG)_4_, 2Ψ(CUG)_4_ and 4Ψ(CUG)_4_). (**D**) Melting curves for (CUG)_4_ RNAs modified with 2′O methyl groups. The total change in melting temperature between unmodified (CUG)_4_ and 4–2′Ome(CUG)_4_ is ∼7°C (62±2, 62±1 and 66±1°C for 1–2′Ome(CUG)_4_, 2–2′Ome(CUG)_4_ and 4–2′Ome(CUG)_4_).

Structure-stabilizing modifications are commonly found in RNAs such as tRNA, rRNA and spliceosomal snRNAs (25–27). Pseudouridine (Ψ) is an isomerization product of uridine and is the most common RNA modification (Figure 1A). It is known to play many important roles in many cellular processes (27). Ψ has been shown to stabilize RNA structural components via base-stacking interactions and water-bridging interactions (28,29). Another common RNA modification found in many cellular RNAs is the replacement of the hydrogen on the 2′-hydroxyl group with a methyl group and is known as 2′-O-methylation (Figure 1B) (30). Due to steric hindrance, 2′-O-methylation induces A-form helices in RNA and is also known to function as a stabilizing element in tRNA and rRNA (31).

Ψ and 2′-O-methyl modifications were introduced into CUG repeats to determine if these nucleotides stabilize the CUG repeats in a helical conformation. Both modifications do indeed increase the thermal stability of model CUG repeat RNAs and reduce MBNL1′s affinity for model CUG repeats. A crystal structure of CUG repeats with a single Ψ substitution showed a water molecule bridging the Ψ–U non-canonical base pair. Molecular dynamics (MD) simulations support the model that this water bridge reduces the dynamic nature of the Ψ–U pair in helical CUG repeats. HeLa cells treated with *in vitro*-transcribed CUG repeat RNA caused mis-splicing of MBNL1 targets. Replacing uridine with Ψ in the CUG repeats rescued MBNL1-mediated splicing events. Furthermore, we found that incorporation of Ψ into a toxic CUG repeat RNA in a zebrafish model of DM1 ameliorated defects in motor function and enhanced viability.

## MATERIALS AND METHODS

Thermal melts were carried out in 20 mM PIPES buffer at a pH of 7.0 and 150 mM NaCl. The buffer was de-gassed before diluting RNA to a concentration of 2 μM. The temperature was raised by 1°C per minute over a range of 25–95°C, and absorbance at 260 nm was monitored by a Cary UV/Vis spectrophotometer. Absorbance was normalized by subtracting the absorbance at 25°C.

GST-tagged MBNL1 (amino acids 2–260) was expressed in Arctic Express *Escherichia coli* cells (Agilent Technologies^TM^) grown to an OD of 0.4, induced with 25 mM IPTG and shaken for 20 h at 16°C. GST-MBNL1 was initially purified over a Hi-TrapGST column on a Fast Protein Liquid Chromatography (FPLC) instrument. MBNL1 was then cleaved from GST by *PreScission Protease* and purified further by affinity chromatography over a Hi-TrapHeparin column. Both columns and the protease were made by GE Healthcare Life Sciences^TM^. MBNL1 concentration was measured by a Bradford assay and the protein was stored in a buffer of 500 mM NaCl, 50% glycerol, 25 mM Tris at pH of 7.5 and 5 mM BME at –80°C until used.

RNA synthesized by Dharmacon was radiolabeled with α-^32^P phosphate with a polynucleotide kinase and stored at –20°C in 20 mM Tris, pH 7.5. Prior to the binding reaction, CUG helices were diluted and folded by incubation at 95°C in 20 mM Tris, pH 7.5, 150 mM NaCl and 5 mM MgCl_2_ for 2 min, followed by incubation on ice for 5 min. The RNA was then incubated with varying MBNL1 concentrations in 175 mM NaCl, 20 mM Tris, pH 7.5, 1 mM BME, 10% glycerol, 5 mM MgCl_2_, 0.1 mg/ml heparin, 2 mg/ml BSA, 0.02% xylene cyanol and 0.05% bromophenol blue. The incubation was 20 min at room temperature. RNA–protein complexes were separated from free RNA on a 6% native acrylamide gel. Complex formation was quantified by exposure on a phosphorimager screen followed by analysis with *ImageQuant* from GE Healthcare Life Sciences^TM^. Affinity constants were then calculated with the following equation: *f_bound_* = *f_max_*([MBNL1]/([MBNL1+*K_D_*)). The images of the gels were enhanced equally across each gel using Adobe Photoshop. No specific feature was enhanced.

The trCUG-3(Ψ5) construct was purchased from Dharmacon and deprotected per the manufacturer's instructions. The RNA was then purified using HPLC and brought to a final concentration of 0.5 mM in a solution of 15 mM NaCl, 5 mM Tris (pH 7.5) and 5 mM MgCl_2_. The RNA was annealed by heating to 70°C for 5 min and rapidly cooled to 4°C. The best crystals grew from a mixture of 2 μL of RNA solution and 2 μL of well solution containing 4 mM MgSO_4_, 50 mM Tris (pH 8.5) and 30% (w/v) 1,6-hexanediol. Crystals appeared in ∼1 week.

Crystals were mounted in rayon loops and flash frozen in liquid nitrogen. Experimental data were collected at the Structural Biology Center at Argonne National Laboratory on the 19ID beamline under a cryostream. The X-ray data were integrated, merged and scaled using the HKL-2000 program suite (32) and converted to structure factors using the CCP4i GUI (33) for the CCP4 program suite (34). Data collection statistics are listed in Table 2.

**Table 1.** Melting Temperatures and MBNL1 affinities for (CUG)_4_ RNAs

RNA	*T_m_* (°C)	MBNL *K_D_*(μM)
(CUG)_4_	59+/−1	0.30 +/− 0.1
1Ψ(CUG)_4_	67+/−2	1.0 +/− 0.3
2Ψ(CUG)_4_	72+/−1	6.0 +/− 2.0
4Ψ(CUG)_4_	78+/−1	>5
1–2′Ome(CUG)_4_	62+/−2	0.65 +/− 0.2
2–2′Ome(CUG)_4_	62+/−1	0.89 +/− 0.2
4–2′Ome(CUG)_4_	66+/−1	1.0 +/− 0.2

**Table 2.** Summary of crystallography data collection and refinement statistics

Measurement	Value		
Space group	R3		
Unit cell dimensions a, b, c (Å)	69.70	69.70	67.80
α, β, γ (°)	90	90	120
Resolution range, Å	22.60–1.90		
Total number of reflections	46959		
Number of unique reflections	9697		
Average redundancy	5.0		
% completeness	96.9 (89.6)		
*I/σI*	22.7 (2.4)		
*R*_*merge*_*^a^*	0.051 (0.44)		
Average B-factors [no. of atoms]			
nucleotides	52.32 [744]		
solvent	55.783 [65]		
*R_free_^b^*	0.267		
*R_work_^b^*	0.196		

Values in parentheses represent highest resolution shell.

^a^$R_{{\rm merge}} = \frac{{\sum {\left| {I - \left\langle I \right\rangle } \right|} }}{{\sum {\left| {\left\langle I \right\rangle } \right|} }}$ where *I* is the observed intensity and <*I>* is the average of intensities obtained from multiple observations of symmetry-related reflections.

^b^$R_{{\rm factor}} = \frac{{\sum {\left| {|F_{\rm o} | - |F_{\rm c} |} \right|} }}{{\sum {|F_{\rm o} |} }}$ where *F*_o_ and *F*_c_ are the observed and calculated structure amplitudes, respectively.

The structure was determined using molrep (35), a part of the CCP4 program suite (34), using the Protein Data Bank (PDB) entry 4FNJ, the structure of trCUG-3 (23), as the search model. The model was rebuilt using Coot (36) and refined with refmac (37). Refinement statistics are listed in Table 2. Coot and PyMOL [Schrodinger, LLC (2010) The PyMOL Molecular Graphics System, Version 1.5.0.1.] were used to generate figures. Simulated annealing omit maps were created using the software suite called Crystallography and NMR System (CNS) (38) by deleting the Ψ5–U31 base pair as well as the coordinating water while all other nucleotides remained fixed during annealing. The structure was deposited in the PDB as entry 4PCJ. Structural parameters were computed using 3DNA (39).

Double-stranded (CUG)_5_ starting structures with 0, 1 and 2 Ψ modifications at the central base pair were constructed from helical crystal structures of CUG repeats (PDB ID: 4FNJ). All simulations were performed utilizing the GROMACS (40–43) MD package on the ACISS cluster at the University of Oregon, using an AMBER (44) force-field optimized for stem-loop RNA structures with modified force-field parameters for the Ψ base (45). All simulations were performed in explicit solvent utilizing the spc/e water model and 150 mM NaCl with excess sodium ions to neutralize the system, Ewald summation for the electrostatics, standard force-field cutoffs and parameters, and in the canonical NVT ensemble, where moles (N), volume (V) and temperature (T) are conserved. A 1 fs time step was utilized, along with standard energy minimization and equilibration techniques, with over 5 ns of equilibration before collecting production data.

After equilibration, water-bridge occupancy was measured using the water-bridge collective variable of the PLUMED software package (46). This collective variable is defined by a numerical function which assigns a continuous measurement to the number of solvent atoms which are within 2 Å of an atom belonging to the A strand U8/Ψ8 base and also within 2 Å of an atom belonging to the pairing B strand U8/ Ψ8 base. This numerical function is rather lengthy and we refer the reader to the PLUMED documentation for further details. Statistics were collected over 10 ns of simulation.

To obtain the free energy in the center-of-mass base separation distance, first an approximate opening pathway was obtained by using the PLUMED (46) implementation of Parrinello's metadynamics algorithm (47) to jointly bias the number of base-pairing contacts and the center-of-mass base separation. Twenty images were chosen along this pathway, anchored using a harmonic restraining potential, from which umbrella sampling runs of 1 ns each were performed. Final calculation of the free energy along the distance coordinate was performed using the weighted historgram analysis method (48) (Grosfield, Alan, ‘WHAM: the weighted histogram analysis method’, version 2.09, http://membrane.urmc.rochester.edu/content/wham).

(CUG)_54_ was transcribed off of 300 ng of a linearized plasmid template containing a T7 promoter upstream of 54 CTG repeats. The reaction conditions were as follows: 0.1 M DTT, 40 mM Tris, pH 8.0, 8 mM MgCl_2_, 50 mM NaCl, 2 mM spermidine 1 mM ATP, 1 mM CTP, 1 mM GTP, 1 mM UTP/ΨTP, 1 μL RNasin (Ambion) and 1 μL T7 polymerase in a final volume of 30 μL. Reactions were incubated at 37°C for 2 h followed by addition of 3.3 μL of RQ1 DNase Buffer and 1 μL RQ1 DNase (Promega) and a 1 h incubation at 37°C. Transcribed RNA was purified by the addition of 80 μL of a 3.75 M LiCl, 25 mM EDTA solution and an overnight incubation at –20°C. The RNA was then pelleted by centrifugation at 13 000 RPM and the liquid was removed. The pellets were washed in 1 mL of 70% ethanol, followed by re-pelleting and re-suspension in 30 μL of ddH_2_O. RNA concentrations were measured on a NanoDrop UV/vis spectrophotometer.

Reporter minigenes, DMPK(CTG)_960_, DMPK(CTG)_40_ and *in vitro*-transcribed RNA were transfected using Lipofectamine 2000 (Life Technologies). Control plasmids were added to transfections containing only reporter minigenes such that each set of cells received 1000 ng of nucleic acid. HeLa cells were routinely cultured as a monolayer in Dulbecco's modified Eagle's medium (DMEM)-GlutaMax medium (Invitrogen) supplemented with 10% fetal bovine serum (Gibco) and 1x antibiotic/antimycotic at 37°C under 5% CO_2_. Prior to transfection, cells were plated in six-well plates at a density of 2.0 × 10^6^ cells per well. Cells were transfected 24 h later at ∼390% confluence using 500 μL of Opti-MEM (Life Technologies) and 5 μL of Lipofectamine 2000. After 4 h, Opti-MEM was replaced with DMEM. Cells were harvested in-plate after 24 h using the RLT buffer from QIAgen's RNeasy kit.

Total RNA was isolated with the RNeasy kit per the manufacturer's instructions. RNA samples were treated with RQ1 DNase and exon inclusion levels were analyzed by reverse transcription and PCR amplification as performed previously in this group (49). Images of the gels were inverted and enhanced equally across each gel using Adobe Photoshop. No specific feature was enhanced.

Zebrafish experiments were conducted as described (50). Briefly, embryos were isolated after paired mating of AB zebrafish (zFIN, Eugene, OR) and injected at the 1–2-cell stage using a Drummond Nanoject. Diluted *in vitro*-transcribed capped and polyadenylated mRNA (4.6 nL each) was injected at a concentration of 100 ng/μL for all constructs unless otherwise specified (approximate total amount of RNA injected/embryo = 0.46 ng). Survival was determined by measuring the number of intact embryos after injection for each genotype and then determining the number of viable embryos at 24 hpf. For each injected RNA, as least three independent experiments were conducted, with at least two separate injection batches conducted per injected RNA at each experiment. The survival% was pooled across all studies and the 95% confidence interval was calculated using the method of Newcombe (51). Fisher exact test was used to compare groups.

Spontaneous coiling was measured as previously described (52,53). Briefly, sets of five embryos injected with the indicated RNAs were collected at 24 hpf and observed over four different 15-s periods. The number of coiling movements per minute per embryo was calculated. *N*>100/genotype from three independent experiments. A one-way Kruskal Wallis ANOVA across genotypes was performed with post-hoc unpaired Mann–Whitney U tests conducted on pre-specified comparisons (Green Fluorescent Protein versus CUG91 and CUG91 vesus Ψ CUG91).

Fluorescence *in-situ* hybridization was carried out on HeLa cells transfected using the same procedure described above. Sixteen hours after transfection, cells were fixed in 4% paraformaldehyde for 15 min. Cells were then permeabilized with 0.5% triton X-100, in 1X PBS at room temperature (RT) for 5 min. Cells were prewashed with 30% formamide, 2X SSC for 10 min at RT. Cells were then probed overnight at 37°C, with a 1 ng/μL of Cy3 (CAG)_10_ probe (IDT, IA) in 30% formamide, 2X SSC, 2 μg/mL BSA, 66 μg/mL yeast tRNA, 2 mM vanadyl complex. Cells were then washed for 30 min in 30% formamide, 2X SSC at 42°C, and then with 1X SSC for 30 min at RT. Cells were mounted onto glass slides using the hard-set mounting media that contains DAPI (Vectashield). Cells were imaged on an Olympus Fluoview FV1000 with a Bx61 scope. Unpaired, two-tailed t-tests were performed using Graphpad's Prism 6. Images were prepared using ImageJ and Adobe Photoshop and the number of foci were quantified using ImageJ's object counter. Large foci were quantified with ImageJ as well.

## RESULTS

### Pseudouridine and 2′-O-methyl modifications increase the thermal stability of short (CUG)_4_ stem-loops

A thermal melting assay was used to determine if Ψ and 2′-O-methyl modifications stabilize a short, helical (CUG)_4_ RNA (Figure 1B). The modifications were targeted to the uridines because these were proposed as dynamic mismatches (23). (CUG)_4_ was synthesized with one, two and four Ψs in place of uridines (Figure 1B). Native (CUG)_4_ melted at 59°C, while the analogs with one, two and four Ψs melted at 67, 72 and 78°C, respectively (Figure 1C, Table 1). (CUG)_4_ RNAs containing one, two and four 2′-O-methyl modification were studied as well (Figure 1B). The *T_m_* increased to 62, 62 and 66°C with one, two and four 2′-O-methyl modifications. These data indicate that Ψ has a more pronounced effect on stabilization of CUG repeats in comparison to 2′-O-methylation.

### MBNL1 has a reduced affinity for pseudouridylated and 2′-O-methylated (CUG)_4_

**Figure 2.**
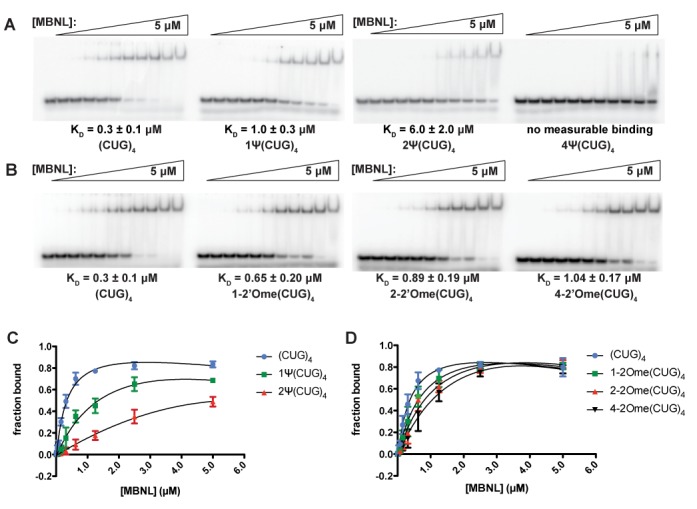
Binding gels and curves for MBNL1-(CUG)_4_ RNA interactions. (**A**) Binding gels from MBNL1-(CUG)_4_ affinity assays for RNAs modified with Ψ. RNAs were radiolabeled with α-P^32^ and combined with different concentrations of a consensus MBNL1 sequence (amino acids 2–260). RNA–protein complex formation induces a band-shift due to an increase in size. *K_D_*s were calculated based on the signal from the radiolabeled RNA and values are approximately the [MBNL1] when 50% of the RNA is bound. (**B**) Binding gels from MBNL1-(CUG)_4_ affinity assays for RNAs modified with 2-’O-methylation. (**C**) Binding curves for (CUG)_4_ RNAs modified with Ψ. Data were fit to the following equation to determine *K_D_* values: *f_bound_* = *f_max_*([MBNL1]/([MBNL1 + *K_D_*)). (**D**) Binding curves for (CUG)_4_ RNAs modified with 2′-O-methylation. *K_D_* values were calculated using the above equation.

The RNAs shown in Figure 1B were used in gel-shift assays to determine how Ψ and 2′-O-methyl modifications affected MBNL1′s binding affinity for the (CUG)_4_ RNA. MBNL1 binds to (CUG)_4_ with a relatively strong affinity (*K_D_* = 0.3 μM). The substitutions of one and two Ψs decreased MBNL1′s affinity for (CUG)_4_, resulting in *K_D_*s for these RNAs of 1.0 and 6.0 μM. Four Ψ replacements in (CUG)_4_ reduced MBNL1′s affinity to such an extent that the *K_D_* was immeasurable in the assay used (Figure 2A, C and Table 1).

Modifying (CUG)_4_ with 2′-O-methylation also reduced MBNL1 binding such that one, two and four methyl modifications increased the *K_D_* to 0.7, 0.9 and 1.0 μM, respectively (Figure 2B, D, Table 1). While 2′-O-methylation did decrease MBNL1′s affinity for CUG repeats, the effect was weaker than that of Ψ. These data are consistent with our model that increasing the stability of the double-stranded, helical conformation of the CUG repeats reduces or eliminates MBNL binding to CUG repeats in this conformation.

### A water molecule bridges the Ψ–U mismatch in a crystal structure and MD simulations

**Figure 3.**
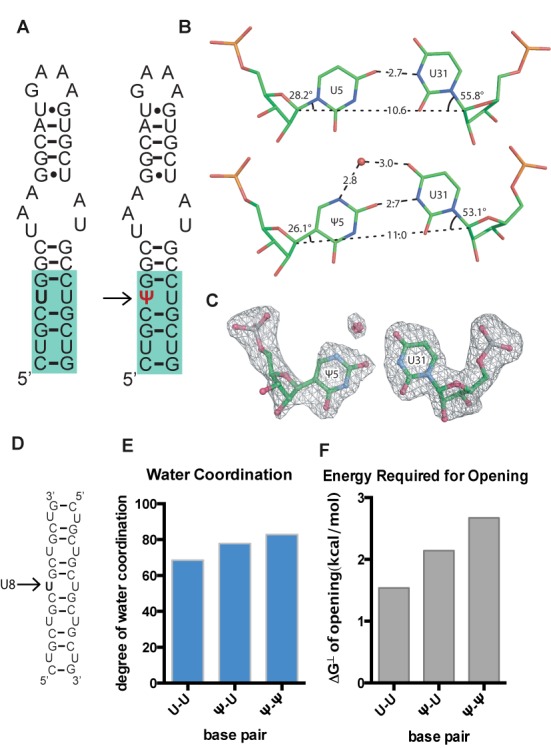
A structure of CUG repeats with an incorporated Ψ and water occupancy through an MD simulation. (**A**) Sequence and predicted secondary structure of constructs used in crystallography. CUG repeats (highlighted in blue) are attached to a GAAA tetraloop/receptor and the Ψ substitution is highlighted in red. trCUG-3 is the native structure and tr-CUG-3(Ψ5) has the Ψ substitution. (**B**) Contacts made in the U5–U31 non-canonical base pair from the native crystal structure compared to the Ψ5–U31 base pair determined in this structure. A bridging water coordinates the amine group of Ψ5 and the carbonyl group of U31 in the Ψ–U base pair. The water bridge introduces two additional hydrogen bonds. (**C**) Simulated annealing omit map (Fo − Fc) of non-canonical Ψ–U pair and bridging water contoured at 5σ. (**D**) (CUG)_5_ RNA used in MD simulations. Prior to running the simulation, RNAs were annealed into dsRNA. Ψ was substituted at the central uridine (U8) on either one or both (CUG)_5_ strands. (**E**) Average degree of water occupancy throughout an MD simulation in U–U, Ψ–U and Ψ–Ψ base pairs. Water occupancy is determined based on the distances between solvent molecules and bases through the simulation. A water molecule is considered to be bridging if it makes contacts with the U and Ψ simultaneously. Plotted here is the average water occupancy normalized to an ideal water bridge. Average degree of water coordination values are as follows: 68.5% for U–U, 77.8% for Ψ–U and 82.9% for Ψ–Ψ. (**F**) Free energy required to open central base pairs. Values represent the average energy required to open U–U, Ψ–U and Ψ–Ψ base pairs throughout MD simulations. Total change in free energy of opening from U–U to Ψ–Ψ is ∼1.5 kcal/mol.

We previously utilized the GAAA tetraloop and its conserved 11 nucleotide receptor in order to facilitate the crystallization of CUG repeats (trCUG-3, Figure 3A) (23). In order to observe how Ψ affected the structure of CUG repeats, we substituted a Ψ in place of the U at position five in the trCUG-3 construct to create trCUG-3(Ψ5) (Figure 3A). The trCUG-3(Ψ5) construct readily crystallized and the structure was successfully solved using the trCUG-3 structure for molecular replacement (see Table 2 for data collection and refinement statistics).

This new structure of trCUG-3(Ψ5) at 1.90 angstroms resolution is nearly identical to the native trCUG-3 structure. Comparison of helical parameters of the CUG portion of the lower helices showed two analogous, primary A-form helices (see Supplementary Table S1 for helical parameters). However, there were key differences when the U5–U31 base pair from the native structure was compared to the Ψ5–U31 base pair (Figure 3B). Strikingly, there was a water molecule coordinating the amine group of Ψ5 to the carbonyl group of U31. Positions of the water and the non-canonical Ψ–U pair were confirmed by creating a simulated annealing omit map (Figure 3C). In order to form these two additional hydrogen bonds, the Ψ5–U31 base pair has a greater opening angle compared to U5–U31 (Supplementary Table S2) as well as a greater C1′–C1′ distance (Figure 3B). This bridging water adds two additional hydrogen bonds between uridine and Ψ and may contribute to Ψ's structure-stabilizing capabilities in the context of CUG repeats.

To assess the potential importance of this bridging water molecule in the Ψ–U base pair, we used MD simulations to study the structure of CUG repeats containing Ψ-substitutions. MD simulations allowed us to investigate how Ψ substitutions affected water coordination and stability of an internal U–U base pair in a simulated solution. The systems investigated *in silico* were hybridized (CUG)_5_ duplex RNA with zero, one and two Ψ-substitutions at the middle (U8–U8) base pair (Figure 3D). Simulations clearly displayed the presence of bridging water molecules, consistent with the crystal structure. RNA with Ψ is much more likely to adopt structures with bridging water molecules. It also showed how the likelihood for RNA of adopting secondary structures mediated by bridging water molecules increases with one and two Ψ substitutions. Bridging water molecules were counted by looking for any identifying solvent molecules that made contact with both pairing bases simultaneously. In a typical 10 ns run of an MD simulation, the average degree of water coordination in a U–U mismatch throughout an MD simulation was 68.5% with respect to the water bridge. This value increased to 77.8% and 82.9% in Ψ–U and Ψ–Ψ mismatches, respectively (Figure 3E).

We also used *in silico* approaches to study the energy required to break open U–U, Ψ–U and Ψ–Ψ base pairs in MD simulations. As expected, pseudouridylated base pairs required more energy to open. The Ψ–Ψ required an additional 1.5 kcal/mol more energy to open compared to the U–U base pair (Figure 3F). Here we have shown that CUG repeats containing Ψ are not only able and likely to form a water bridge, the presence of the water appears to contribute to structural stability.

### *In vitro*-transcribed CUG repeats disrupt MBNL-mediated alternative splicing and the effects are rescued by Ψ incorporation

After determining that Ψ substitutions stabilized the formation of secondary structure in the CUG repeat RNA and prevented MBNL1 binding, we next asked how pseudouridylation of CUG repeats would affect MBNL-mediated splicing events in a cell model. The significant effect on MBNL1 binding to CUG repeats suggested that pseudouridylated CUG repeats would not sequester MBNL proteins and the proteins would be free to regulate alternative splicing. To test this hypothesis, *in vitro*-transcribed CUG repeats with and without Ψ modifications were transiently transfected into HeLa cells along with MBNL-regulated splicing targets.

We used the T7 transcription system to generate (CUG)_54_ RNA with 0%, 50% and 100% Ψ content. The T7 polymerase produced equivalent quantities of RNAs with UTP and ΨTP, suggesting that this enzyme efficiently incorporated the Ψ base. After cleanup, these RNAs were co-transfected into HeLa cells with two different mini-genes containing MBNL1-regulated exons. The *TNNT2* and *INSR* mini-genes were used to monitor the effect of pseudouridylation on the ability of (CUG)_54_ RNA to alter splicing. A plasmid [DMPK(CTG)_960_] that had previously been shown to significantly alter MBNL-regulated splicing was used as a positive control (7), as well as a plasmid [DMPK(CTG)_40_] with fewer repeats for comparison to the T7-produced (CUG)_54_ RNA.

**Figure 4.**
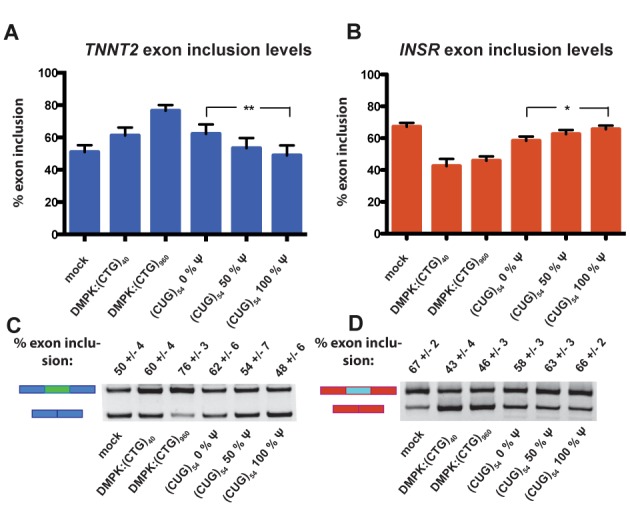
CUG repeats with increasing levels of Ψ are unable to induce mis-splicing of MBNL1-regulated targets *TNNT2* and *INSR.* (**A**) *TNNT2* exon inclusion levels from a HeLa cell-splicing assay. The sample labeled mock represents percent exon inclusion when the reporter construct is transfected. The samples labeled DMPK:(CTG)_40_ and DMPK:(CTG)_960_ have the% exon inclusion when *TNNT2* reporter as well as a plasmid containing the DMPK 3′UTR _with_ 40 and 960 CTG repeats were transfected. The next three samples are the exon inclusion levels when the reporter along with *in vitro*-transcribed CUG repeats with increasing levels of Ψ were transfected. The difference in exon inclusion between the 0% Ψ samples and the 100% Ψ samples was statistically significant via a student's t test with a *P* = 0.0021 (** indicates that *P* ≤ 0.01). (**B**) *INSR* exon inclusion levels from a HeLa cell-splicing assay. These assays were set up following the same experimental theme described above, using an *INSR* reporter minigene instead of *TNNT2*. *P* = 0.0239 (* indicates that *P* ≤ 0.05). (**C**) A representative gel from the *TNNT2* HeLa cell-splicing assay. Upper bands represent spliced transcripts with exons included and lower bands represent spliced transcripts with exons excluded. Quantification of these bands was used to obtain percent exon inclusion. (**D**) A representative gel from the *INSR* HeLa cell-splicing assay.

The basal level of exon 5 inclusion for *TNNT2* splicing reporter alone (mock) was 50% due to the activity of endogenous MBNL proteins in the HeLa cells (Figure 4A and C). Transfecting the *TNNT2* reporter as well as DMPK(CTG)_40_ and DMPK(CTG)_960_ resulted in 60% and 76% exon inclusion due to sequestration of MBNL proteins and a loss of activity. MBNL proteins promote exclusion of exon 5 of *TNNT2* (11), so cellular expression of CUG repeats resulted in an increased level of exon inclusion as expected. Cells transfected with the *in vitro-*transcribed (CUG)_54_ with 0%, 50% and 100% Ψ altered the *TNNT2* exon inclusion levels to 62%, 54% and 48%, respectively (Figure 4A and C). The 62% level of exon 5 inclusion is comparable to 60% exon 5 inclusion observed with the DMPK(CTG)_40_ plasmid, suggesting these two approaches resulted in similar levels of CUG repeats, and thus MBNL sequestration, in the cells. Ψ incorporation into (CUG)_54_ resulted in reversion to the basal level of exon inclusion (50%), presumably because MBNL proteins were no longer sequestered to the CΨG repeats and they were free to regulate *TNNT2* exon 5 inclusion.

To determine if pseudouridylation was able to reduce or eliminate the change in splicing of another MBNL-mediated event, we measured exon inclusion levels of *INSR*. For this splicing event, MBNL1 promotes inclusion of the regulated exon, exon 11 (10). HeLa cells transfected with only the *INSR* reporter had an exon inclusion level of 67%, and co-transfection with DMPK(CTG)_40_ and DMPK(CTG)_960_ resulted in 43% and 46% exon 11 inclusion (Figure 4B and D). Transfection of (CUG)_54_ with 0% Ψ resulted in 58% exon 11 inclusion, an effect which is partially rescued by 50% Ψ (63% exon 11 inclusion) and nearly fully rescued by 100% Ψ (66% exon 11 inclusion) (Figure 4B and D). These results are consistent with pseudouridylation of the CUG repeats reducing or eliminating the ability of the modified RNA to sequester MBNL proteins in this DM1 cell model.

### Pseudouridylated CUG repeats form reduced-sized foci

Previous studies have shown that extended CUG repeats form nuclear foci in DM1 tissues, animal and cell culture models (54). To determine whether pseudouridylated RNA is capable of forming RNA foci, we performed fluorescence in-situ hybridization, probing for CUG repeats with a Cy-3 labeled (CAG)_10_ probe. HeLa cells were transfected with (CUG)_54_ RNA transcribed with 0%, 50% and 100% Ψ, as well as plasmids containing the DMPK 3′UTR with 40 and 960 CTG repeats as described above for the splicing assay. Cells transfected with an unrelated plasmid (mock) formed 1.3 ± 0.6 foci/cell, while those transfected with DMPK(CTG)_40_ and DMPK(CTG)_960_ formed 21 ± 11 and 34 ± 12 foci/cell, respectively. When we transfected cells with (CUG)_54_ RNA with 0%, 50% and 100% Ψ they formed 19 ± 10, 15 ± 6 and 20 ± 7 foci/cell, respectively, and there was no significant difference between unmodified and fully modified transfected RNA (Supplementary Figure S1 and Table S3). However, cells transfected with fully modified CUG repeats tended to form smaller foci than those transfected with unmodified RNA. Large foci were deemed those with a volume larger than 0.2 μm^3^. The cells transfected with unmodified CUG repeats had 69 ± 17% large foci, while those transfected with fully modified CUG repeats had 47 ± 17% large foci. The difference in percent-large-foci between unmodified and modified RNA is significant with a *P* value <0.0001 (Supplementary Figure S1 and Table S3).

### Pseudouridylation suppresses CUG repeat-associated toxicity in a zebrafish model of myotonic dystrophy

**Figure 5.**
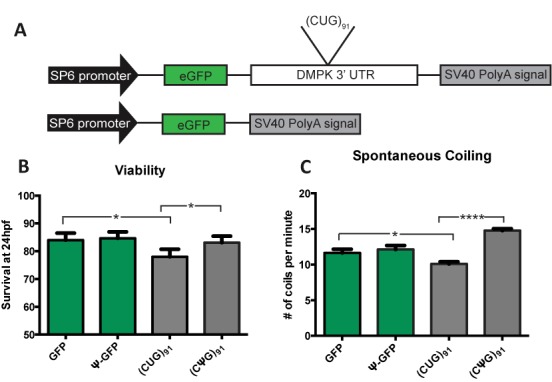
Viability and motoric function of zebrafish embryos injected with CUG repeat RNA is modified by pseudouridylation. (**A**) DMPK and GFP control constructs injected into zebrafish. These RNAs were transcribed *in vitro* with either all UTP or all Ψ-NTP. RNA containing the DMPK 3′ UTR has 91 CUG repeats. (**B**) Viability of zebrafish embryos at 24 h post fertilization after injection with *in vitro-*transcribed GFP or GFP:(CUG)_91_ RNA containing either uridine or Ψ. There was no significant difference in the viability of embryos injected with GFP with and without Ψ. Embryos injected with (CUG)_91_ RNA transcribed *in vitro* with 100% Ψ exhibit significantly enhanced viability compared to uridine-containing (CUG)_91_ RNA. *N*>400/>500 zebrafish per genotype over at least three independent experiments. For GFP versus (CUG)_91_ the *P* value was 0.014. For RNA (Ψ(CUG)_91_ versus (CUG)_91_ the *P* value was 0.030. *P* < 0.05 by Fishers exact test. (**C**) Spontaneous coiling movements in 24 hpf zebrafish embryos injected with the indicated *in vitro-*transcribed RNAs. Coiling data is graphed as the number of spontaneous coiling movements/minute. (CUG)_91_ RNA-injected embryos exhibit impaired early muscle development and function, with decreased spontaneous coiling at 24 hpf. In contrast, embryos injected with 100% Ψ (CUG)_91_ RNA exhibit normal spontaneous coiling rates compared to GFP-injected embryos (*P* < 0.0001). *N*>100/ zebrafish per genotype over three independent experiments were analyzed. All groups were significantly different by a Kruskal Wallis ANOVA. * indicates *P* < 0.0001 by Post-hoc Mann-Whitney U test for viability. * indicates *P* < 0.05 and **** indicates *P* < 0.0001 by standard unpaired, two-tailed t test for spontaneous coiling.

To assess the *in vivo* effects of Ψ incorporation on CUG repeat-associated toxicity, we utilized an established zebrafish model of DM1 (50). This model utilizes *in vitro*-transcribed RNA encoding GFP fused with the 3′UTR of DM1, which contains 91 CUG repeats. When injected into single cell embryos, this CUG RNA triggers alterations in morphology, viability and motor behaviors within the first 24 h of embryo development (Figure 5B and C) (50). To assess the impact of pseudouridylation on CUG repeat toxicity *in vivo*, transcribed GFP-(CUG)_91_ RNAs with Ψ-NTP were injected into zebrafish embryos. We used GFP alone-encoding mRNA lacking CUG expansions with uridine or Ψ as a control (Figure 5A). RNA levels post-injection were measured using qRT-PCR to determine whether pseudouridylated RNA has a similar stability in the embryos and there was no significant difference between levels (Supplementary Figure S2). As previously described (50), injection of GFP-(CUG)_91_ mRNA into embryos led to a significant decrease in viability at 24 h post-fertilization (hpf) compared to embryos injected with an equimolar amount of GFP RNA [GFP versus (CUG)_91_] (Figure 5B) (50). In contrast, when embryos were injected with GFP-(CUG)_91_ RNA transcribed with 100% Ψ-NTP, survival at 24 hpf was significantly enhanced compared to 0% Ψ-NTP GFP-(CUG)_91_ and was similar to embryos injected with GFP RNA (Supplementary Figure S5b).

**Figure 6.**
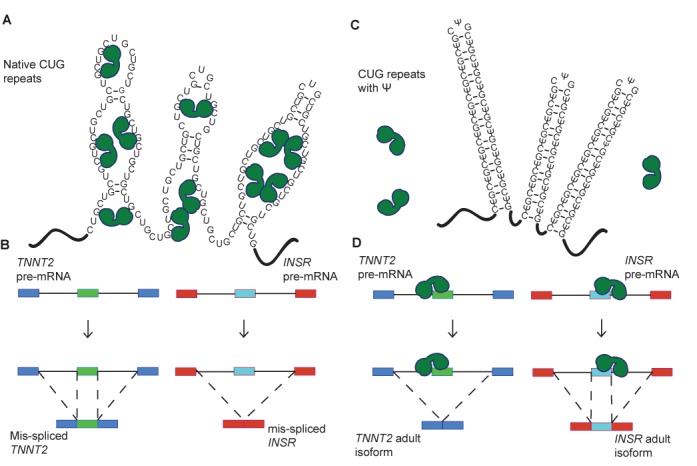
A model for CUG repeat detoxification via pseudouridylation. (**A**) Native CUG repeats form unstable A-form helices that are proposed to adopt multiple conformations, including partially unstructured RNA. Partially unfolded CUG repeats present the Watson–Crick faces of numerous MBNL1 binding sites (YGCY). In this case, MBNL1 binds to the CUG repeats and is sequestered to their location in the nucleus. MBNL1 is represented by green shapes. (**B**) When MBNL1 is sequestered to CUG repeats, it is unavailable to bind to the pre-mRNAs that it regulates. Here CUG repeats increased exon inclusion in the *TNNT2* transcript, which is characteristic of the fetal isoform. CUG repeats result in decreased exon inclusion in the *INSR* mRNA in the presence of CUG repeats, also characteristic of the fetal isoform. (**C**) CUG repeats with Ψ substitutions (CΨG repeats) form stable stem-loop structures. In these structures the Watson–Crick face of MBNL1′s binding site is unavailable for interaction and MBNL1 is not sequestered. (**D**) MBNL1 is free to bind to its target transcripts when CΨG repeats are present, thus exon inclusion levels for the regulated exons in *TNNT2* and *INSR* are wild-type.

Zebrafish models of neuromuscular disorders often exhibit abnormalities in basic motor behaviors during early development (50,53). The first observable indication of skeletal muscle activity is spontaneous coiling, the alternating contraction of trunk and tail that begins at 17 hpf, peaks at 19 hpf and then decreases over the next 8 h (55). At 24 hpf, zebrafish embryos injected with CUG repeats displayed a defect in spontaneous coiling [GFP: 12 ± 0.52 coils/min, (CUG)_91_:10 ± 0.30 coils/min, *P* = 0.022] (Figure 5C) (50). However, pseudouridylation of the CUG RNA completely abolished this toxicity, resulting in an increase in coiling activity [Ψ (CUG)_91_:15 ± 0.26 coils/min, *P* < 0.0001] (Figure 6B). These results indicate that substituting Ψ for uridine in CUG repeats reduces the toxicity of the RNA as demonstrated by the rescue in viability and rescue (enhanced activity) of coiling activity.

## DISCUSSION

Structural and biochemical studies on MBNL1 with RNA substrates indicated that this family of proteins prefers to bind single-stranded or partially structured RNA (24). This leads us to hypothesize that stabilizing the CUG repeats in a helical conformation with RNA modifications would decrease or eliminate MBNL binding to this toxic RNA. Of two RNA modifications tested, we found Ψ significantly stabilized the CUG repeats in a helical (A-form) conformation, and, as hypothesized, this stabilization of CUG repeats inhibited MBNL1 binding (Figures 1 and 2). Furthermore, in a DM1 cell model, pseudouridylation of CUG repeats reduced and eliminated the ability of the CUG repeats to cause mis-splicing of two MBNL1 splicing reporters (Figure 4). Significantly, pseudouridylation of a CUG repeat-containing mRNA protected zebrafish embryos from increased morbidity and decreased spontaneous coiling compared to mRNA without the Ψ modification (Figure 5). These studies are consistent with the model shown in Figure 6 that depicts pseudouridylation stabilizing CUG repeats in a helical conformation and not sequestering MBNL proteins (Figure 6C and D) while unmodified CUG repeats are less stable (or more prone to partial unfolding) and MBNL proteins are sequestered by the repeats, and mis-splicing results (Figure 6A and B). While CUG repeats have been shown to form stable stem-loop structures via structure-probing assays, we have shown that Ψ induces further stabilization, preventing MBNL1 binding (20).

Past efforts to understand the mechanism through which Ψ stabilizes RNA structure indicated that this modification leads to stronger base-stacking interactions with surrounding nucleotides and increased hydrogen bonding contacts through coordination with water molecules (28). According to our analyses, the coordination of the water molecule induced formation of two extra H-bonds in the Ψ–U base pair compared to the U–U base pair (Figure 3B and C). Although this water in the crystal structure could be due to crystal packing, the MD studies with a CUG RNA containing Ψ showed a significant increase in the degree of water coordination in this same position compared to the unmodified CUG RNA (Figure 3D–F). It is likely that Ψ stabilizes RNA structure through different mechanisms and this new mechanism adds to the repertoire (28).

Our results are consistent with CAG antisense and small molecule approaches that stabilize the CUG repeats in helical structures (no mismatches in CAG-CUG antisense interactions) or through binding of molecules to the CUG repeats in helical conformation (56–58). However, CAG antisense and small molecule approaches do not address the secondary structure of RNA. Because modifying CUG repeats to stabilize structure prevents MBNL binding, we have shown that the secondary structure of the RNA is important in the DM1 disease mechanism. Furthermore, these data are congruent with approaches that stabilize CUG repeats in helical conformations and specific targeting of the U–U mismatches. SnoRNAs have been used to target RNAs with some promising results for altering RNA structure and function in cells (59,60). To significantly pseudouridylate CUG repeats *in vivo* with snoRNAs would be quite challenging because of the abundance of sites and lack of specificity for specific uridines within the repeats, therefore this avenue is not currently being pursued.

More broadly, our results suggest that the conformation of toxic RNAs should be considered for disease mechanisms and therapeutic strategies. For example, the CCUG repeats that cause myotonic dystrophy type 2 can adopt multiple RNA structures including possibly unfolded when protein-bound, partially unfolded, and two different helical structures with different mismatches (16,61). The expanded CGG repeats in the 5′ UTR of the fragile X mental retardation 1 gene results in FTAXS, a disease characterized by ataxia, tremors and neurodegeneration. Extended expansion of these repeats results in Fragile X Syndrome (FXS), a common form of mental retardation (62). RNA-binding proteins associate with helical CGG repeats and are sequestered to nuclear inclusions (63). Spinocerebellar ataxia 8 (SCA8) is caused by expansion of CTG repeats in the *ataxin* 8 gene, and the resulting RNA gain of function produces cerebellar atrophy and coordination defects (64,65). Expanded CUG repeats in SCA8 could exhibit the same pathogenic mechanism as that in DM1 and DM2, and RNA modifications could be used to help guide therapeutic strategies. SCA10 and ALS/FTD are autosomal dominant neurodegenerative diseases that are caused by expanded penta- and hexanucleotide repeats, and in both cases RNA-binding proteins are sequestered to repeats, resulting in mis-regulation of splicing (66–68). The RNA modification strategy described here is a powerful approach to address the conformation of RNA repeats associated with human diseases that should be considered for disease mechanism and therapeutic targeting.

## SUPPLEMENTARY DATA

Supplementary Data are available at NAR Online.

SUPPLEMENTARY DATA

## References

[1] O'Rourke J.R., Swanson M.S. (2009). Mechanisms of RNA-mediated disease. J. Biol. Chem..

[2] Cho D.H., Tapscott S.J. (2007). Myotonic dystrophy: emerging mechanisms for DM1 and DM2. Biochim. Biophys. Acta.

[3] Lee J.E.J., Cooper T.A.T. (2009). Pathogenic mechanisms of myotonic dystrophy. Biochem. Soc. Trans..

[4] Miller J.W.J., Urbinati C.R.C., Teng-Umnuay P.P., Stenberg M.G.M., Byrne B.J.B., Thornton C.A.C., Swanson M.S.M. (2000). Recruitment of human muscleblind proteins to (CUG)(n) expansions associated with myotonic dystrophy. EMBO J..

[5] Mankodi A., Urbinati C.R., Yuan Q.P., Moxley R.T., Sansone V., Krym M., Henderson D., Schalling M., Swanson M.S., Thornton C.A. (2001). Muscleblind localizes to nuclear foci of aberrant RNA in myotonic dystrophy types 1 and 2. Hum. Mol. Genet..

[6] Pascual M., Vicente M., Monferrer L., Artero R. (2006). The Muscleblind family of proteins: an emerging class of regulators of developmentally programmed alternative splicing. Differentiation.

[7] Goers E.S., Purcell J., Voelker R.B., Gates D.P., Berglund J.A. (2010). MBNL1 binds GC motifs embedded in pyrimidines to regulate alternative splicing. Nucleic Acids Res..

[8] Warf M.B., Nakamori M., Matthys C.M., Thornton C.A., Berglund J.A. (2009). The protein factors MBNL1 and U2AF65 bind alternative RNA structures to regulate splicing. Proc. Natl Acad. Sci. U.S.A..

[9] Machuca-Tzili L., Brook D., Hilton-Jones D. (2005). Clinical and molecular aspects of the myotonic dystrophies: a review. Muscle Nerve.

[10] Savkur R.S., Philips A.V., Cooper T.A. (2001). Aberrant regulation of insulin receptor alternative splicing is associated with insulin resistance in myotonic dystrophy. Nat. Genet..

[11] Philips A.V., Timchenko L.T., Cooper T.A. (1998). Disruption of splicing regulated by a CUG-binding protein in myotonic dystrophy. Science.

[12] de Die-Smulders C.E., Höweler C.J., Thijs C., Mirandolle J.F., Anten H.B., Smeets H.J., Chandler K.E., Geraedts J.P. (1998). Age and causes of death in adult-onset myotonic dystrophy. Brain.

[13] Ho T.H., Charlet-B N., Poulos M.G., Singh G., Swanson M.S., Cooper T.A. (2004). Muscleblind proteins regulate alternative splicing. EMBO J..

[14] Cass D., Hotchko R., Barber P., Jones K., Gates D.P., Berglund J.A. (2011). The four Zn fingers of MBNL1 provide a flexible platform for recognition of its RNA binding elements. BMC Mol. Biol..

[15] Fu Y., Ramisetty S.R., Hussain N., Baranger A.M. (2011). MBNL1-RNA recognition: contributions of MBNL1 Sequence and RNA Conformation. Chem. Biol. Chem..

[16] Warf M.B., Berglund J.A. (2007). MBNL binds similar RNA structures in the CUG repeats of myotonic dystrophy and its pre-mRNA substrate cardiac troponin T. RNA.

[17] Yuan Y., Compton S.A., Sobczak K., Stenberg M.G., Thornton C.A., Griffith J.D., Swanson M.S. (2007). Muscleblind-like 1 interacts with RNA hairpins in splicing target and pathogenic RNAs. Nucleic Acids Res..

[18] Du H., Cline M.S., Osborne R.J., Tuttle D.L., Clark T.A., Donohue J.P., Hall M.P., Shiue L., Swanson M.S., Thornton C.A. (2010). Aberrant alternative splicing and extracellular matrix gene expression in mouse models of myotonic dystrophy. Nat. Struct. Mol. Biol..

[19] Kanadia R.N., Shin J., Yuan Y., Beattie S.G., Wheeler T.M., Thornton C.A., Swanson M.S. (2006). Reversal of RNA missplicing and myotonia after muscleblind overexpression in a mouse poly (CUG) model for myotonic dystrophy. Proc. Natl. Acad. Sci. U.S.A..

[20] Napierała M., Krzyzosiak W.J. (1997). CUG repeats present in myotonin kinase RNA form metastable ‘slippery’ hairpins. J. Biol. Chem..

[21] Mooers B.H.M., Logue J.S., Berglund J.A. (2005). The structural basis of myotonic dystrophy from the crystal structure of CUG repeats. Proc. Natl. Acad. Sci. U.S.A..

[22] Kiliszek A., Kierzek R., Krzyzosiak W.J., Rypniewski W. (2009). Structural insights into CUG repeats containing the ‘stretched U-U wobble’: implications for myotonic dystrophy. Nucleic Acids Res..

[23] Coonrod L.A., Lohman J.R., Berglund J.A. (2012). Utilizing the GAAA tetraloop/receptor to facilitate crystal packing and determination of the structure of a CUG RNA helix. Biochemistry.

[24] Teplova M., Patel D.J. (2008). Structural insights into RNA recognition by the alternative-splicing regulator muscleblind-like MBNL1. Nat. Struct. Mol. Biol..

[25] Newby M.I., Greenbaum N.L. (2001). A conserved pseudouridine modification in eukaryotic U2 snRNA induces a change in branch-site architecture. RNA.

[26] Ofengand J., Bakin A., Wrzesinski J., Nurse K., Lane B.G. (1995). The pseudouridine residues of ribosomal RNA. Biochem. Cell Biol..

[27] Charette M., Gray M.W. (2000). Pseudouridine in RNA: what, where, how, and why. IUBMB life.

[28] Davis D.R. (1995). Stabilization of RNA stacking by pseudouridine. Nucleic Acids Res..

[29] Newby M.I., Greenbaum N.L. (2002). Investigation of Overhauser effects between pseudouridine and water protons in RNA helices. Proc. Natl. Acad. Sci. U.S.A..

[30] Ji L., Chen X. (2012). Regulation of small RNA stability: methylation and beyond. Cell Res..

[31] Kawai G., Yamamoto Y., Kamimura T., Masegi T., Sekine M., Hata T., Iimori T., Watanabe T., Miyazawa T., Yokoyama S. (1992). Conformational rigidity of specific pyrimidine residues in tRNA arises from posttranscriptional modifications that enhance steric interaction between the base and the 2′-hydroxyl group. Biochemistry.

[32] Otwinowski Z., Minor W. (1997). Processing of X-ray diffraction data collected in oscillation mode. Macromol. Crystallogr. Pt A.

[33] Potterton E., Briggs P., Turkenburg M., Dodson E. (2003). A graphical user interface to the CCP4 program suite. Acta Crystallogr. D Biol. Crystallogr..

[34] Winn M.D., Ballard C.C., Cowtan K.D., Dodson E.J., Emsley P., Evans P.R., Keegan R.M., Krissinel E.B., Leslie A.G.W., McCoy A. (2011). Overview of the CCP4 suite and current developments. Acta Crystallogr. D Biol. Crystallogr..

[35] Vagin A., Teplyakov A. (1997). MOLREP: an automated program for molecular replacement. J. Appl. Crystallogr..

[36] Emsley P., Lohkamp B., Scott W.G., Cowtan K. (2010). Features and development of Coot. Acta Crystallogr. D Biol. Crystallogr..

[37] Murshudov G.N., Vagin A.A., Dodson E.J. (1997). Refinement of macromolecular structures by the maximum-likelihood method. Acta Crystallogr. D Biol. Crystallogr..

[38] Brünger A.T., Adams P.D., Clore G.M., DeLano W.L., Gros P., Grosse-Kunstleve R.W., Jiang J.S., Kuszewski J., Nilges M., Pannu N.S. (1998). Crystallography & NMR system: a new software suite for macromolecular structure determination. Acta Crystallogr. D Biol. Crystallogr..

[39] Lu X.-J., Olson W.K. (2008). 3DNA: a versatile, integrated software system for the analysis, rebuilding and visualization of three-dimensional nucleic-acid structures. Nat. Protoc..

[40] Berendsen H.J., van der Spoel D., van Drunen R. (1995). GROMACS: a message-passing parallel molecular dynamics implementation. Comput. Phys. Commun..

[41] Lindahl E., Hess B., van der Spoel D. (2001). GROMACS 3.0: a package for molecular simulation and trajectory analysis. Mol. Model. Annu..

[42] van der Spoel D., Lindahl E., Hess B., Groenhof G., Mark A.E., Berendsen H.J.C. (2005). GROMACS: fast, flexible, and free. J. Comput. Chem..

[43] Hess B., Kutzner C., van der Spoel D., Lindahl E. (2008). GROMACS 4: algorithms for highly efficient, load-balanced, and scalable molecular simulation. J. Chem. Theory Comput..

[44] Zgarbová M., Otyepka M., Sponer J., Mládek A., Banáš P., Cheatham T.E., Jurečka P. (2011). Refinement of the Cornell et al. nucleic acids force field based on reference quantum chemical calculations of glycosidic torsion profiles. J. Chem. Theory Comput..

[45] Aduri R., Psciuk B.T., Saro P., Taniga H., Schlegel H.B., SantaLucia J. (2007). AMBER force field parameters for the naturally occurring modified nucleosides in RNA. J. Chem. Theory Comput..

[46] Bonomi M., Branduardi D., Bussi G., Camilloni C., Provasi D., Raiteri P., Donadio D., Marinelli F., Pietrucci F., Broglia R.A. (2009). PLUMED: a portable plugin for free-energy calculations with molecular dynamics. Comput. Phys. Commun.

[47] Laio A., Parrinello M. (2002). Escaping free-energy minima. Proc. Natl. Acad. Sci. U.S.A..

[48] Kumar S., Rosenberg J.M., Bouzida D., Swendsen R.H., Kollman P.A. (1992). THE weighted histogram analysis method for free-energy calculations on biomolecules. I. The method. J. Comput. Chem..

[49] Purcell J., Oddo J.C., Wang E.T., Berglund J.A. (2012). Combinatorial mutagenesis of MBNL1 zinc fingers elucidates distinct classes of regulatory events. Mol. Cell. Biol..

[50] Todd P.K., Ackall F.Y., Hur J., Sharma K., Paulson H.L., Dowling J.J. (2014). Transcriptional changes and developmental abnormalities in a zebrafish model of myotonic dystrophy type 1. Dis. Model. Mech..

[51] Newcombe R.G. (1998). Two-sided confidence intervals for the single proportion: comparison of seven methods. Stat. Med..

[52] Dowling J.J., Low S.E., Busta A.S., Feldman E.L. (2010). Zebrafish MTMR14 is required for excitation-contraction coupling, developmental motor function and the regulation of autophagy. Hum. Mol. Genet..

[53] Telfer W.R., Busta A.S., Bonnemann C.G., Feldman E.L., Dowling J.J. (2010). Zebrafish models of collagen VI-related myopathies. Hum. Mol. Genet..

[54] Taneja K.L., McCurrach M., Schalling M., Housman D., Singer R.H. (1995). Foci of trinucleotide repeat transcripts in nuclei of myotonic dystrophy cells and tissues. J. Cell Biol..

[55] Brustein E., Saint-Amant L., Buss R.R., Chong M., McDearmid J.R., Drapeau P. (2003). Steps during the development of the zebrafish locomotor network. J. Physiol. Paris.

[56] Wheeler T.M., Leger A.J., Pandey S.K., MacLeod A.R., Nakamori M., Cheng S.H., Wentworth B.M., Bennett C.F., Thornton C.A. (2012). Targeting nuclear RNA for in vivo correction of myotonic dystrophy. Nature.

[57] Childs-Disney J.L., Hoskins J., Rzuczek S.G., Thornton C.A., Disney M.D. (2012). Rationally designed small molecules targeting the RNA that causes myotonic dystrophy type 1 are potently bioactive. ACS Chem. Biol..

[58] Jahromi A.H., Nguyen L., Fu Y., Miller K.A., Baranger A.M., Zimmerman S.C. (2013). A novel CUGexp· MBNL1 inhibitor with therapeutic potential for myotonic dystrophy type 1. ACS Chem. Biol..

[59] Zhao X., Yu Y.-T. (2007). Targeted pre-mRNA modification for gene silencing and regulation. Nat. Meth..

[60] Stepanov G.A., Semenov D.V., Kuligina E.V., Rabinov O.K.I., Kit Y.Y., Richter V.A. (2012). Analogues of artificial human box C/D small nucleolar RNA as regulators of alternative splicing of a pre-mRNA target. Acta Naturae.

[61] Childs-Disney J.L., Yildirim I., Park H., Lohman J.R., Guan L., Tran T., Sarkar P., Schatz G.C., Disney M.D. (2014). Structure of the myotonic dystrophy type 2 RNA and designed small molecules that reduce toxicity. ACS Chem. Biol..

[62] Jacquemont S., Hagerman R.J., Leehey M., Grigsby J., Zhang L., Brunberg J.A., Greco C., Des Portes V., Jardini T., Levine R. (2003). Fragile X premutation tremor/ataxia syndrome: molecular, clinical, and neuroimaging correlates. Am. J. Hum. Genet..

[63] Kiliszek A., Kierzek R., Krzyzosiak W.J., Rypniewski W. (2011). Crystal structures of CGG RNA repeats with implications for fragile X-associated tremor ataxia syndrome. Nucleic Acids Res..

[64] Day J.W., Schut L.J., Moseley M.L., Durand A.C., Ranum L.P. (2000). Spinocerebellar ataxia type 8: clinical features in a large family. Neurology.

[65] Daughters R.S., Tuttle D.L., Gao W., Ikeda Y., Moseley M.L., Ebner T.J., Swanson M.S., Ranum L.P.W. (2009). RNA gain-of-function in spinocerebellar ataxia type 8. PLoS Genet..

[66] Matsuura T., Yamagata T., Burgess D.L., Rasmussen A., Grewal R.P., Watase K., Khajavi M., McCall A.E., Davis C.F., Zu L. (2000). Large expansion of the ATTCT pentanucleotide repeat in spinocerebellar ataxia type 10. Nat Genet.

[67] Wakamiya M., Matsuura T., Liu Y., Schuster G.C., Gao R., Xu W., Sarkar P.S., Lin X., Ashizawa T. (2006). The role of ataxin 10 in the pathogenesis of spinocerebellar ataxia type 10. Neurology.

[68] DeJesus-Hernandez M., Mackenzie I.R., Boeve B.F., Boxer A.L., Baker M., Rutherford N.J., Nicholson A.M., Finch N.A., Flynn H., Adamson J. (2011). Expanded GGGGCC hexanucleotide repeat in noncoding region of C9ORF72 causes chromosome 9p-linked FTD and ALS. Neuron.

