# Supplementary material for: Modifications to toxic CUG RNAs induce structural stability, rescue mis-splicing in a myotonic dystrophy cell model and reduce toxicity in a myotonic dystrophy zebrafish model
Source: Nucleic Acids Res. 2014 Oct 10;42(20):12768–78. doi: 10.1093/nar/gku941 (PMC4227782; doi:10.1093/nar/gku941)
Supplement: SUPPLEMENTARY DATA [file supp_42_20_12768__index.html]

Modifications to toxic CUG RNAs induce structural stability, rescue mis-splicing in a myotonic dystrophy cell model and reduce toxicity in a myotonic dystrophy zebrafish model — Modifications to toxic CUG RNAs induce structural stability, rescue mis-splicing in a myotonic dystrophy cell model and reduce toxicity in a myotonic dystrophy zebrafish model — SUPPLEMENTARY DATA 

# Modifications to toxic CUG RNAs induce structural stability, rescue mis-splicing in a myotonic dystrophy cell model and reduce toxicity in a myotonic dystrophy zebrafish model

## SUPPLEMENTARY DATA

**Files in this Data Supplement:**

- SUPPLEMENTARY DATA
