# Supplementary material for: Modifications to toxic CUG RNAs induce structural stability, rescue mis-splicing in a myotonic dystrophy cell model and reduce toxicity in a myotonic dystrophy zebrafish model
Source: Nucleic Acids Res. 2014 Oct 10;42(20):12768–78. doi: 10.1093/nar/gku941 (PMC4227782; doi:10.1093/nar/gku941)
Supplement: SUPPLEMENTARY DATA [file supp_gku941_nar-01242-y-2014-File009.pdf]

Supplementary Figure 1

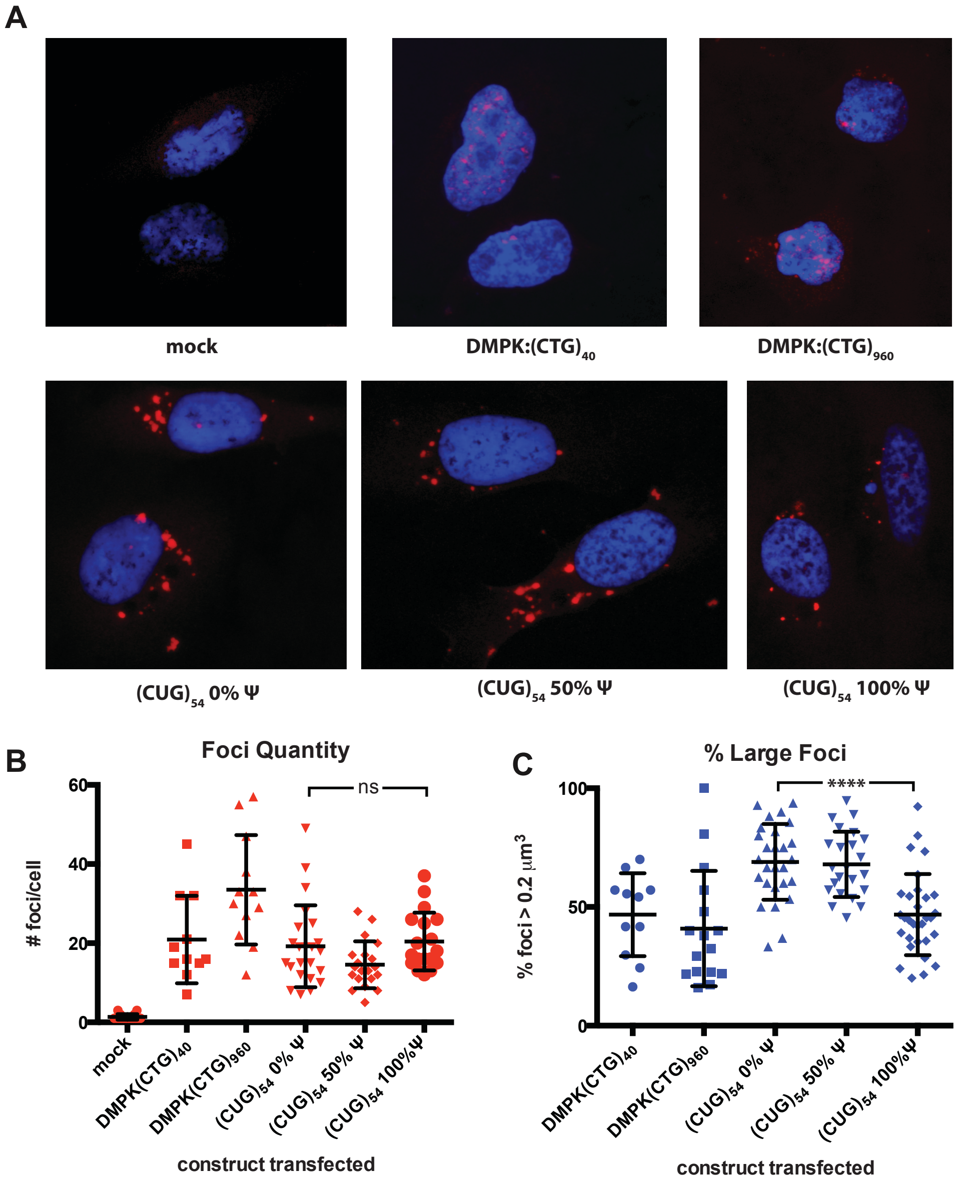

Supplementary Figure 2

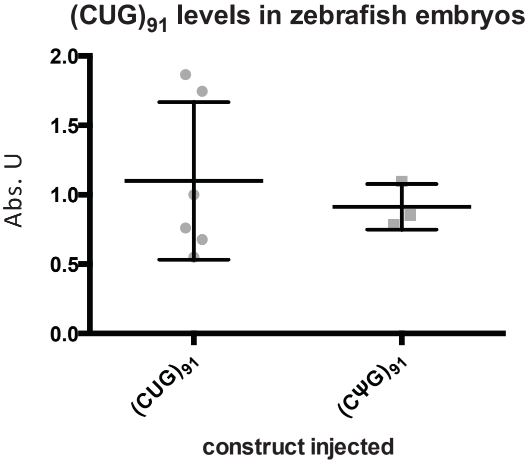

Supplemental Table 1. Comparison of CUG repeat helical parameters

	Roll	Twist	Slide	Rise	Inclination	Helical twist	x-displacement	Helical rise	Zp	Zp(h)
**B DNA[1]**	0.60	36.00	0.23	3.32	2.10	36.50	0.05	3.29	-0.36	-0.02
**A DNA[1]**	8.00	31.10	-1.53	3.31	14.70	32.50	-4.17	2.83	2.24	4.19
**(CUG)2**	9.17	33.48	-1.34	3.10	18.57	35.89	-4.70	2.39	2.35	4.62
**(CUG)2(Ψ5)**	10.09	33.68	-1.34	3.17	20.05	36.09	-4.53	2.44	2.23	4.74

[1]A and B-form structural parameters from: Lu, X.-J., & Olson, W. K. (2003). 3DNA: a software package for the analysis, rebuilding and visualization of three-dimensional nucleic acid structures. Nucleic acids research, 31(17), 5108–5121.

Supplemental Table 2. Comparison of U-U and Ψ-U non-canonical base pairs.

		C1'-C1'	1st hbond	2nd hbond	λ I	λ II	Incline[1]	Shear	Stretch	Stagger	Buckle	Propeller	Opening	Type
**trCUG-3**	**U2-U34**	8.6	2.8	2.8	44.8	75.9	minor	-2.47	-1.89	0.26	-7.12	-16.4	11.6	Type I
	**U5-U31**	10.6	2.7	-	28.2	55.8	minor	-2.63	-1.33	-0.1	5.62	-7.12	-25.98	Type II
**trCUG-3(Ψ5)**	**U2-U34**	8.7	2.8	2.9	44.5	73.0	minor	-2.26	-1.87	0.15	-0.62	-18.37	10.22	Type I
	**Ψ5-U31**	11.0	2.7	-	26.1	53.1	minor	-3.00	-1.17	-0.34	7.48	-6.69	-38.77	Type II

Classification	# of hbonds	Incline
**Type I**	2	minor
**Type II**	1	minor
**Type III**	0	none
**Type IV**	1	major
**Type V**	2	major
**Type VI**	0	major

[1]Direction of incline was determined by considering the difference between λI and λII. If (λI – λII) < -8, then the pair was considered to be inclined toward the minor groove; however, if the difference was >8, the pair was considered to be inclined toward the major groove. Any values between -8 and 8 were considered to be not inclined.

Supplemental Table 3: quantification of CUG foci

construct	# foci	% lg foci (0.2 um^3)
mock	1.3 ± 0.6 N=16	N/A
DMPK:(CTG)40	21 ± 11 N=11	47 ± 17 N=11
DMPK:(CTG)960	34 ± 12 N=12	41 ± 24 N=16
(CUG)54 0%	19 ± 10 N=23	69 ± 17 N=27
(CUG)54 50%	15 ± 6 N=20	68 ± 14 N=23
(CUG)54 100%	20 ± 7 N=18	47 ± 17 N=31

Supplementary Figure 1: CUG repeat foci in HeLa cells. (A) Images of HeLa cells transfected with mock plasmid, plasmids containing 40 and 960 CTG repeats in the DMPK gene as controls, as well as cells transfected with $(CUG)_{54}$ RNA with 0%, 50% and 100% Ψ. Plasmid controls expressed nuclear foci, while cells transfected with RNA expressed cytoplasmic foci. (B) Cells transfected with 960 CTG repeats tended to form more foci, while those transfected with 40 CTG repeats and modified and unmodified RNA formed similar numbers of foci. (C) Modified RNA did induce formation of foci that tended to be smaller than 0.2 μm^3.

Supplementary Figure 2: CUG repeat levels in zebrafish are independent of modification. CUG repeat levels were measured in zebrafish embryos injected with unmodified and fully pseudouridylated $(CUG)_{91}$. The method used to measure RNA levels was qRT-PCR using primers specific for the injected transcript. Modified and unmodified levels of RNA were insignificantly different ($p=0.61$).
